# Optimal body weight for health and longevity: bridging basic, clinical, and population research

**DOI:** 10.1111/acel.12207

**Published:** 2014-03-14

**Authors:** Luigi Fontana, Frank B Hu

**Affiliations:** 1 Division of Geriatrics and Nutritional Science, Washington UniversitySt.Louis, MO, USA; 2 Department of Clinical and Experimental Sciences, Brescia University Medical SchoolBrescia, Italy; 3 CEINGE Biotecnologie AvanzateNapoli, Italy; 4 Departments of Nutrition and Epidemiology, Harvard School of Public HealthBoston, MA, USA; 5 Channing Laboratory, Department of Medicine, Brigham and Women’s Hospital and Harvard Medical SchoolBoston, MA, USA

**Keywords:** body mass index, calorie intake, disease prevention, health, obesity

## Abstract

Excess body weight and adiposity cause insulin resistance, inflammation, and numerous other alterations in metabolic and hormonal factors that promote atherosclerosis, tumorigenesis, neurodegeneration, and aging. Studies in both animals and humans have demonstrated a beneficial role of dietary restriction and leanness in promoting health and longevity. Epidemiological studies have found strong direct associations between increasing body mass index (BMI) and risks of developing type 2 diabetes, cardiovascular disease, and several types of cancer, beginning from BMI of 20–21 kg m^−2^. Although a recent meta-analysis suggests that overweight individuals have significantly lower overall mortality than normal-weight individuals, these data are likely to be an artifact produced by serious methodological problems, especially confounding by smoking, reverse causation due to existing chronic disease, and nonspecific loss of lean mass and function in the frail elderly. From a clinical and public health point of view, maintaining a healthy weight through diet and physical activity should remain the cornerstone in the prevention of chronic diseases and the promotion of healthy aging.

## Introduction

Worldwide, an estimated 2.16 billion adults and 170 million children are overweight [body mass index (BMI) > 25 kg m^−^²] or obese (BMI > 30 kg m^−^²) (Lobstein *et al*., 2004; Finucane *et al*., 2011). In the United States, an alarming 67% and 33% of the adults are overweight and obese, respectively (Flegal *et al*., 2012). Data from animal and human studies clearly indicate that excess adiposity, especially if centrally located, is associated with a range of detrimental metabolic alterations that increase the risk of developing type 2 diabetes, coronary artery disease, hypertension, stroke, nonalcoholic liver disease, cancer, neurodegeneration, and accelerated aging (Tchernof & Després, 2013). Starting from a BMI of 20–21 kg m^−2^, rising BMI levels are associated with progressively increased risks of developing diabetes, cardiovascular disease (CVD), and several types of cancer, which combined are responsible for approximately two-thirds of deaths in Western countries (Clinical, 1998).

According to the World Health Organization and many medical societies, BMI should be maintained in the 18.5–24.9 kg m^−2^ range to achieve optimum health. However, a recent meta-analysis has suggested that being overweight, and possibly even mildly obese, is associated with a reduced mortality risk (Flegal *et al*., 2013). These findings clearly contradict evidence from basic scientific, clinical, and epidemiological studies on the metabolic benefits of maintaining a healthy weight. They also are inconsistent with data from dietary restriction (DR) studies in rodents and primates as well as basic research studies on the metabolic and molecular mechanisms promoting health and longevity. In this paper, we review and address the effects of overweight and obesity on health and longevity by integrating evidence from basic, clinical, and population-based studies. We will also discuss the clinical and public health implications of these data.

### Body mass index and mortality

The relationship between body weight and mortality has been the subject of debate for several decades. In particular, considerable controversy has surrounded the shape of the curve for the association between BMI and mortality, and the effects of overweight on mortality. In a recent meta-analysis of 97 prospective cohort studies (2.9 million individuals and 270 000 deaths), Flegal *et al*. found that compared with the normal-weight group (18.5–<25 kg m^−2^), the relative risks (RRs) of death were 0.94 (95% CI, 0.91–0.96) for overweight (25–<30), 0.95 (95% CI, 0.88–1.01) for class 1 obesity (30–<35), and 1.29 (95% CI, 1.18–1.41) for class 2 and 3 obesity (≥35) (Flegal *et al*., 2013). However, the validity of these findings has been challenged due to several major methodological problems (Tobias & Hu, 2013). First, many high-quality prospective studies and consortia (including >6 million participants) were excluded from the meta-analyses because they did not use standard BMI categories (i.e., 18.5–24.9 for normal weight, 25–29.9 for overweight, and ≥30 for obesity). These large studies generally benefited from sufficient statistical power to allow for the analysis of finer BMI categories, and therefore had no reason to use such broad categories. In most of these omitted studies, the BMI range associated with the lowest mortality was around 22.5–25, particularly after accounting for smoking status and reverse causation due to prevalent diseases (Tobias & Hu, 2013). Second, the meta-analysis included numerous studies conducted among elderly or sick populations as well as current and past smokers. In particular, the broad reference group (BMI 18.5–24.9) contains not only individuals who are lean and active, but also heavy smokers, the frail and elderly, and those who are ill with previous weight loss or diminished weight gain due to existing diseases. Because the overweight and obese groups were compared with this heterogeneous group, the associations with the higher-BMI groups were seriously underestimated, creating an artifact of reduced mortality among the overweight and moderately obese groups (Willett *et al*., 2013).

Reverse causation (i.e., low BMI being the result of underlying illness rather than the cause) is the most serious concern in analyzing the relationship between BMI and mortality (Hu, 2008). Weight loss can result from the direct effects of chronic disease on weight or from conscious efforts motivated by a diagnosis of serious illness. Many conditions that cause weight loss, such as chronic obstructive pulmonary disease, depression, and neurodegenerative diseases, may remain undiagnosed for years. For example, weight loss in patients with Parkinson’s disease starts 7–8 years before diagnosis (Chen *et al*., 2003). To minimize the impact of reverse causation, it is important to exclude patients with existing chronic diseases at baseline (Hu, 2008). Also, residual confounding by preclinical or undiagnosed conditions may attenuate the effects of obesity on mortality. Excluding deaths during the first few years of follow-up can also reduce bias due to reverse causation, because those with existing or occult diseases tend to die early in follow-up (Hu, 2008).

**Figure 1:**
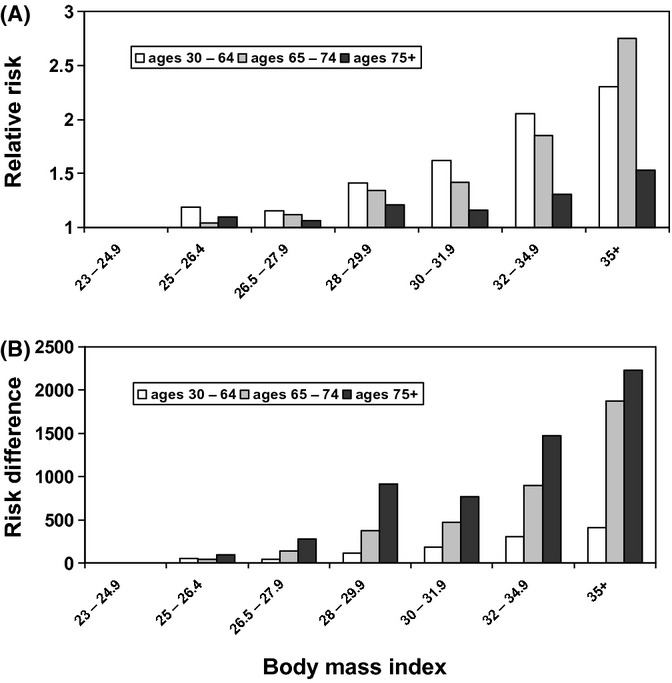
Risk of death associated with body mass index among male nonsmokers without chronic health conditions, according to age. The annual risk of death is expressed as both the RR (Panel A) and the absolute amount of additional risk (risk difference) (Panel B) per 100 000 population, as reported in the American Cancer Society Cancer Prevention Study 2. From: Byers T (2006). Overweight and mortality among baby boomers–now we’re getting personal. N Engl J Med **355**,758–60.

The problem of reverse causation becomes even more pronounced in studies among older individuals as the prevalence of chronic diseases increases with age. Thus, age has been found to be an apparent effect modifier in the association between BMI and mortality, suggesting attenuation in the positive association between BMI and mortality among older age groups. In addition to greater bias due to higher prevalence of chronic diseases in the elderly, BMI is a less valid measure of excess body fat in older populations due to differential loss of muscle mass related to aging (sarcopenia or frailty) (Manson *et al*., 2007). In the Cancer Prevention Study II, stratified analyses by three age groups (30–64 years, 65–74 years, and ≥75 years) showed stronger association of mortality associated with increasing BMI in younger than older participants (Calle *et al*., 1999). However, because overall mortality is much higher in the elderly, the absolute increase in death rates associated with higher BMIs was much greater in people ≥75 years old than in middle-aged individuals (Fig. 1) (Byers, 2006). Thus, the reduced relative impact of obesity on mortality does not mean that obesity is not detrimental in the elderly.

The ‘obesity paradox,’ i.e., the association of obesity with improved survival or decreased mortality, is commonly observed among patients with wasting conditions (e.g., congestive heart failure, end-stage renal disease, advanced malignancies, and AIDS). In these populations, increased blood pressure and serum cholesterol concentrations are also associated with improved survival (Curtis *et al*., 2005). A biological basis for the obesity paradox is unclear; it has been suggested that higher BMI among the chronically ill may confer survival benefits by providing ‘metabolic or nutritional reserve,’ but this hypothesis has yet to be tested. On the other hand, methodological bias from reverse causation is a more plausible explanation, because patients with low BMI, blood pressure, and cholesterol usually have more severe disease and are associated with greater frailty and a more rapid decline in physical health (Hu, 2008). Thus, the mortality risk in the low-BMI group is substantially increased, resulting in artificially low mortality rates in the higher-BMI groups. Another explanation for the obesity paradox is survival bias or depletion of susceptibles, due to more frequent premature deaths among those most vulnerable to obesity-related complications. This bias is exacerbated in short-term follow-up studies.

Confounding by smoking is also a major concern, because smokers tend to weigh less than nonsmokers but have substantially higher mortality rates (Manson *et al*., 1987). Although adjustment for smoking can reduce the impact of confounding, statistical adjustment for smoking status is typically inadequate due to complex variations in duration of smoking and degrees of inhalation. The best way to minimize residual confounding by smoking is to restrict analyses to never-smokers. Because reverse causation and confounding by smoking typically coexist in a population, it is important to simultaneously exclude both persons with known chronic disease and smokers from the analyses to obtain valid estimates.

To account for smoking and reverse causation due to prevalent diseases, two large pooled analyses of individual-level data from more than two million people have been recently published. In the Prospective Studies Collaboration, the analyses were adjusted for age, sex, and smoking status (Whitlock *et al*., 2009). The first five years of follow-up were excluded to limit reverse causation. Mortality was lowest at a BMI of 22.5–25 for both sexes. Above this range, each five-unit increment in BMI was associated with about 29% higher overall mortality ([95% CI 27–32%]); below this range, excess mortality was due mainly to smoking-related diseases. Stratified analysis by smoking status revealed a relatively direct linear relationship among nonsmokers, while a nonlinear J-shaped relationship persisted among smokers.

In the National Cancer Institute Cohort Consortium, which included 19 cohorts and 1.46 million people, the age-standardized rate of death from any cause was lowest among participants with a BMI of 22.5–24 (Berrington de Gonzalez *et al*., 2010). Among female healthy participants who never smoked, with a BMI of 22.5–24.9 as the reference category, RRs of mortality were 1.13 for a BMI of 25.0–29.9; 1.44 for a BMI of 30.0–34.9; 1.88 for a BMI of 35.0–39.9; and 2.51 for a BMI of 40.0–49.9. The results were similar for men. When BMI was analyzed as a continuous variable, the RR for each five-unit increase was 1.31 (95% CI, 1.29–1.33) over the range of 25.0–49.9. The association between BMI and mortality increased with longer duration of follow-up. When stratified by age of BMI ascertainment, the RRs were stronger in the younger age groups (<70 years old) than the older age group (≥70 years).

Taken together, these two large pooled analyses of individual-level data, along with data from several large well-established individual cohort studies, convincingly show that BMI values associated with the lowest mortality were generally below 25, and that being overweight or moderately obese was associated with significantly increased risk of mortality, especially among healthy nonsmokers. Of note, pooled analyses of individual-level data are usually superior to meta-analyses of published estimates, because the former can standardize exposure and covariate definitions, exclusion criteria, and methods for statistical adjustments, and easily facilitate crucial stratified analyses.

### Fat distribution and mortality

Abdominal or central obesity, reflected by a higher waist circumference and waist-to-hip ratio (WHR), has been associated with increased mortality, independent of BMI (Zhang *et al*., 2008). In the NHS cohort, after adjustment for BMI and potential confounders, the RRs across the lowest to the highest waist circumference quintiles were 1.00, 1.11, 1.17, 1.31, and 1.79 (95% CI, 1.47–1.98) for all-cause mortality. Among normal-weight women, abdominal obesity was significantly associated with increased CVD mortality: RR associated with waist circumference ≥ 88 cm was 3.02 (95% CI, 1.31–6.99) and for WHR ≥ 0.88 was 3.45 (95% CI, 2.02–6.92). The strong association between WHR and mortality in normal-weight women suggests that a relatively high degree of abdominal adiposity is deleterious despite being normal weight. These results clearly demonstrate the importance of abdominal obesity in predicting mortality. However, the studies do not diminish the importance of overall adiposity, particularly in younger and middle-aged people due to a strong correlation between BMI and waist circumference (*r* > 0.80) (Hu, 2008).

### BMI and morbidity

**Figure 2:**
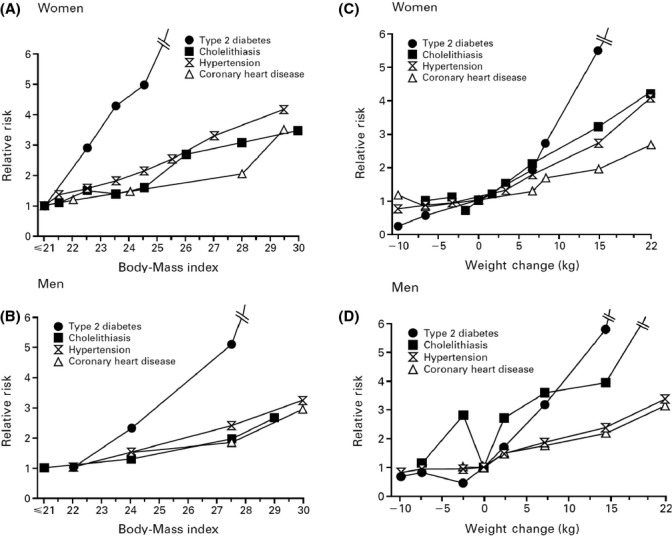
Relation between body mass index up to 30 and the relative risk of type 2 diabetes, hypertension, coronary heart disease, and cholelithiasis in women (Panel A) and men (Panel B). Relation between the change in weight and the relative risk of type 2 diabetes, hypertension, coronary heart disease, and cholelithiasis in women (Panel C) and men (Panel D). From: Willett WC, Dietz WH, Colditz GA (1999). Guidelines for healthy weight. *N Engl J Med*. **341**, 427–434.

Epidemiological studies provide overwhelming evidence that above a BMI of 20–21, a strong and linear association exists between BMI and the risk of developing type 2 diabetes, hypertension, CVD, cholelitiasis, and other chronic diseases in both men and women (Fig. 2A,B) (Willett *et al*., 1999). The strongest magnitude of association is observed for diabetes. There also appears to be a linear relationship between BMI and coronary heart disease (CHD), which is particularly evident among Asian populations. Jee and colleagues published a detailed analysis of BMI and CHD incidence among 133 740 Korean participants during nine years of follow-up (Jee *et al*., 2005). After adjustment for age, sex, and smoking status, each 1-unit increase in BMI was associated with a 14% (95% CI, 12–16%) increased risk of incident CHD. Compared with a BMI of 18–18.9, even a normal BMI of 24–24.9 was associated with a twofold increased risk of CHD.

Significant weight gain from young adulthood through midlife is common and largely reflects increases in body fat. Large cohort studies have found a strong dose–response relationship between the amount of weight gained since young adulthood (age 18 for women and age 20 for men) and risk of developing diabetes, hypertension, CHD, and cholelithiasis (Fig. 2C,D). Even a modest amount of weight gain (<5 kg) since young adulthood was significantly associated with an increased risk of several chronic diseases independent of baseline weight. Greater weight gain (e.g., 11–19 kg) was associated with twofold increased risk of CHD in a cohort of women (Willett *et al*., 1995). Therefore, monitoring weight change since young adulthood can provide a sensitive and useful clinical measure for early detection of adverse trends in chronic disease risk.

Convincing evidence from epidemiological studies indicates that excess adiposity contributes to the increased incidence and/or death from cancers of the colon, female breast (in postmenopausal women), endometrium, kidney (renal cell), esophagus (adenocarcinoma), gastric cardia, pancreas, gallbladder, and liver (Calle & Kaaks, 2004). Compared to normal weight, both overweight and obesity are associated with significantly increased risks of these cancers, attributing to 15–20% of all cancer deaths in the USA (Calle *et al*., 2003).

Body fat distribution, measured by waist circumference or WHR, has also been associated with metabolic disease, CVD, and several major cancers independent of BMI and other risk factors (Hu, 2008). However, including BMI and waist circumference in a model simultaneously tends to attenuate the effects of BMI. It should be noted that, after adjusting for waist circumference, the interpretation of BMI is altered, such that BMI now largely reflects the effects of lean body mass rather than overall adiposity. Nonetheless, measuring waist circumference among normal-weight individuals can provide additional predictive power of disease risk beyond BMI.

Increasing physical fitness or becoming physically active can reduce the adverse impacts of overweight and obesity on health (LaMonte & Blair, 2006). However, physical activity does not completely eliminate the adverse effects of obesity on disease risk. Similarly, being lean does not entirely counteract the increased risk associated with physical inactivity. Individuals who are both lean and physically active tend to have the lowest risk of chronic disease incidence and mortality (Hu *et al*., 2004). These findings indicate that leanness, accompanied or achieved by an active lifestyle, is the optimal way to reduce the risk of chronic diseases, and that excess adiposity remains a concern even among active individuals.

### Optimal weight and successful aging

The concept of successful aging includes not only the absence of major chronic diseases but also the maintenance of sound cognitive, physical, and other functions (Rowe & Kahn, 1997). Traditional cardiovascular risk factors such as smoking and physical inactivity have been associated with a decreased probability of successful aging. However, few studies have examined whether adiposity affects healthful aging among those who survive to older ages. Given that more people survive to older ages, it is critical to identify the optimal body weight to predict healthy aging. In the Hawaii Lifespan Study, a study of aging men from the Honolulu Heart Program/Honolulu-Asia Aging cohort, being overweight in midlife was associated with a significantly reduced probability of healthy survival to age 85 or older among men (Willcox *et al*., 2006). Whether this finding can be generalized to women or other ethnic groups remains unclear.

**Figure 3:**
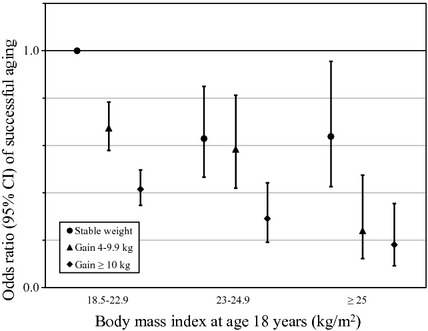
Joint effect of body mass index at age 18 and weight change on healthy survival in the Nurses’ Health Study. Adjusted odds with 95% confidence intervals. Multivariable model adjusted for age (years), education (registered nurse, bachelor, master, or doctoral degree), husband’s education (less than high school, some high school, high school graduate, college graduate, or graduate school), marital status (married, widowed, separated/divorced/never married), postmenopausal hormone use (never used, past user, or current user), smoking status (never smoked, past smoker, current smoker 1–14, 15–24, or ≥25 cigarettes/day), family history of heart disease (yes/no), family history of diabetes (yes/no), family history of cancer (yes/no), vigorous physical activity (hour/week), polyunsaturated–saturated fat ratio (in fifths), intakes of trans fat, alcohol, and cereal fiber (all in fifths), and intakes of fruits and vegetables and red meat (in thirds), all defined at baseline. From Sun Q, Townsend MK, Okereke OI, Franco OH, Hu FB, Grodstein F (2009). Adiposity and weight change in midlife in relation to healthy survival after age 70 in women: prospective cohort study. *BMJ*. **339**, b3796.

Adiposity in early adulthood and midlife has been associated with decreased probability of healthy survival at age 70 or older in the Nurses’ Health Study (NHS) (Sun *et al*., 2009). Healthy survivors were participants who survived to age 70 free from major chronic diseases (cancer, diabetes, CVD, kidney failure, chronic obstructive pulmonary disease, and neurodegenerative disease), had no major impairment of cognitive function, had no major limitation of physical functions, and were in good mental health. In the NHS cohort, approximately 10% of participants were designated ‘healthy survivors.’ After adjustment for multiple potential confounding factors, every one-unit increase in BMI was associated with a 12% reduction in the odds of healthy survival (95% CI 10–14%). Compared with lean women (BMI 18.5–22.9), obese women (BMI ≥ 30) had 79% lower odds of healthy survival to the age of ≥70 (95% CI 71–85%). In addition, weight gain since age 18 was associated with decreased odds of healthy survival after the age of 70 in a dose–response manner (Fig. 3). Even a moderate weight gain of 4–10 kg was significantly associated with reduced odds of healthy survival.

Collectively, this evidence underscores the importance of maintaining a healthy weight throughout adulthood to enjoy a long and healthy life. All three metrics in the ‘adiposity triad’ (Hu, 2008) (BMI, waist circumference, and weight gain since young adulthood) are important in assessing the relationship between adiposity and healthy aging, because each adds information to the risk prediction and offers a unique aspect of potential goals and targets for prevention.

### Linking metabolic pathways to chronic diseases: Fat tissue as an endocrine organ

**Figure 4:**
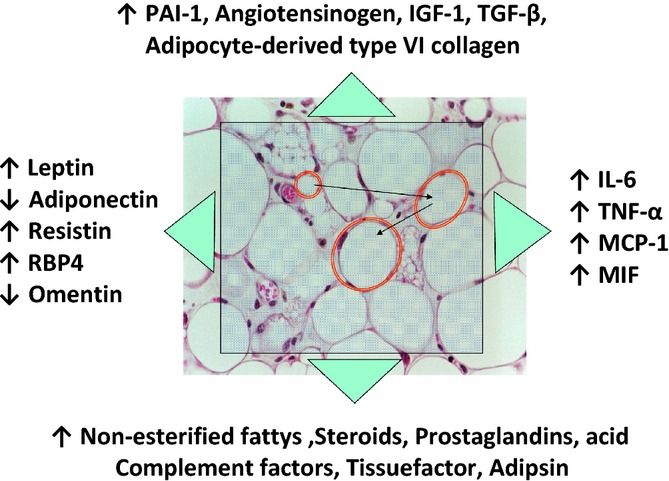
Adipose tissue as an endocrine organ. A chronic positive energy balance induces adipose tissue expansion (red circles) and recruitment of M1 stage macrophages. Enlarged adipocytes and activated macrophages secrete proteins and lipids, called adipokines, that promote inflammation (e.g., IL-6, TNF-alpha, MCP-1, MIF, resistin), insulin resistance (reduced adiponectin and omentin; increased TNF-alpha, leptin, RBP4, resistin, and nonesterified free fatty acids), cell proliferation (leptin, IGF-1, TGF-β, adipocyte-derived type VI collagen), and blood pressure and coagulation dysfunction.

Accumulating evidence indicates that adipose tissue is not just an inert store of excessive energy intake, but is also an active endocrine organ that produces key hormones, called adipokines, that regulate several important biological functions (Fig. 4) (Kershaw & Flier, 2004). Excessive energy intake, particularly in sedentary people, leads to a chronic positive energy balance, resulting in weight gain, increased visceral adiposity, dysfunctional enlarged adipocytes with macrophages infiltration, lipid overflow, and deposition of ectopic fat in key organs, such as liver, heart, skeletal muscle, kidney, and pancreas (Tchernof & Després, 2013). Both hypertrophic fat cells and infiltrated inflammatory cells secrete inflammatory cytokines, including interleukin-6 (IL-6) and tumor necrosis factor (TNF)-α, causing a local and systemic low-grade inflammation. This chronic inflammation is known to play a central role in the pathogenesis of atherosclerosis, cancer, dementia, and aging (Fontana *et al*., 2007a; Gregor & Hotamisligil, 2011). Inflammation and reduced production of adiponectin (an adipocyte-derived adipokine) from enlarged visceral fat cells in overweight and obese individuals are associated with insulin resistance, increased hepatic glucose production, and altered lipoprotein–lipid metabolism (i.e., hypertriglyceridemia, low HDL cholesterol, and small, dense LDL particles), contributing to the development of type 2 diabetes, nonalcoholic fatty liver disease, and CVD (Tchernof & Després, 2013; National Institutes of Health, 1998; Gregor & Hotamisligil, 2011; Turer & Scherer, 2012). Moreover, adipokine-mediated insulin resistance triggers compensatory hyperinsulinemia, which exerts powerful mitogenic procancer effects directly, and also indirectly by increasing the bioavailability of sex hormones and insulin-like growth factor (IGF)-1, and the ovarian production of androgens. Enlarged adipocytes also secrete molecules such as leptin, IGF-1, IL-6, and type VI collagen that promote cell survival and tumor growth (Calle & Kaaks, 2004; Roberts *et al*., 2010). Insulin resistance, hyperinsulinemia, and adipokine imbalance, in conjunction with overactivation of the renin-angiotensin-aldosterone and the sympathetic nervous systems, have also been implicated in the pathogenesis of hypertension in overweight and obese individuals (Tchernof & Després, 2013).

A 5–10% weight loss induced by a negative energy balance (i.e., calorie restriction, endurance exercise, bariatric surgery) simultaneously improves multiple metabolic and hormonal factors implicated in the pathogenesis of several lethal chronic diseases, as well as in the biology of aging itself (Goldstein, 1992). Weight loss induced by a negative energy balance is associated with a reduction in visceral, hepatic, and skeletal muscle fat, decreased fat cell size, increased adiponectin and insulin sensitivity, and reduced circulating levels of insulin and leptin (Larson-Meyer *et al*., 2006; Uusitupa *et al*., 2003; Turer & Scherer, 2012). Weight loss is also associated with improved blood pressure, serum triglycerides, and HDL-cholesterol concentrations. (Dattilo & Kris-Etherton, 1992; Stevens *et al*., 2001). Decreases in adiposity also leads to reductions in inflammatory cytokines and prostaglandins, oxidative stress and DNA damage, and circulating estrogens due to inhibition of aromatases (Davì *et al*., 2002; Ziccardi *et al*., 2002; Hofer *et al*., 2008). Other hormonal adaptations associated with fat loss, improved insulin sensitivity, and reduced cancer risk include increased serum steroid hormone binding globulin and reduced levels of free estrogens and testosterone, and increased serum IGFBP-1 and reduced free IGF-1 concentrations (Longo & Fontana, 2010; Demark-Wahnefried *et al*., 2012).

The beneficial effects of sustained weight loss induced by a negative energy balance have recently been confirmed in nonrandomized, prospective, controlled studies, in which morbidity and mortality from diabetes, CVD, and cancer were dramatically reduced among obese gastric bypass surgery patients (Sjöström *et al*., 2007; Carlsson *et al*., 2012). Nonetheless, experimental studies indicate that a reduction in fat mass alone (i.e., surgical removal of large quantities of subcutaneous abdominal fat or omental fat) without modifications in energy balance does not improve insulin sensitivity, inflammation, or other cardiometabolic risk factors in obese patients (Klein *et al*., 2004; Fabbrini *et al*., 2010), indicating that induction of a negative energy balance is required to achieve the metabolic benefits of weight loss.

### Health benefits of achieving a healthy weight through dietary restriction

It is well established that diet and exercise-induced weight loss improve metabolic and cardiovascular health. Data from experimental studies show that dietary restriction (DR), defined as a reduction in food intake below usual ad libitum intake without malnutrition, and avoiding the excessive energy intake that leads to overweight and obesity, is the most powerful intervention to extend healthy lifespan in mammals (Weindruch & Walford, 1988; Masoro, 2005; Fontana & Klein, 2007). However, the ideal body weight, percent adiposity, and metabolic profile associated with healthy longevity and the lowest risk of developing chronic disease remain unknown. Data from hundreds of studies conducted in simple model organisms and small mammals clearly indicate that restricting caloric intake by 30–50% slows aging and markedly extends average and maximal lifespan (Weindruch & Walford, 1988; Masoro, 2005; Fontana *et al*., 2010). Not only do ‘super-lean’ DR mice and rats live up to 50% longer, but also they are protected against cancer, cardiomyopathy, nephropathy, diabetes, neurodegenerative disease, and chronic lung and autoimmune diseases (Weindruch & Walford, 1988; Masoro, 2005; Fontana & Klein, 2007). Rather than simply prolonging the survival of an old, frail, and ill animal, DR maintains the physiological functions of these mammals in more youthful states compared with age-matched control animals with free access to food. For example, sarcopenia, left ventricular diastolic dysfunction, decline in heart rate variability, presbycusis, and neurodegeneration are prevented or delayed in DR rodents (Marzetti *et al*., 2009; Meyer *et al*., 2006; Mager *et al*., 2006; Someya *et al*., 2010; Martin *et al*., 2006). Additionally, data from postmortem pathological studies have shown that 30% of the DR rodents vs. 6% of fatter ad-libitum-fed animals die in old age free of pathological lesions (Shimokawa *et al*., 1993). Data from two ongoing primate studies show that 30% DR, started at a young age and sustained for 20 years, is safe and results in a more than a 50% reduction in cardiovascular and cancer morbidity and mortality (Colman *et al*., 2009; Mattison *et al*., 2012). In the Wisconsin study, type 2 diabetes and glucose intolerance were completely prevented and age-dependent decline in skeletal muscle mass, auditory function, and brain volume were significantly attenuated in DR monkeys compared with the ad-libitum control group (Colman *et al*., 2008, 2009; Someya *et al*., 2010). In this study, but not in the NIA study, age-related mortality was significantly lower in the DR than in the control monkeys; however, both studies found that the percentage of animals free of age-related diseases was higher in the DR than in the control group (Colman *et al*., 2009; Mattison *et al*., 2012).

A series of randomized, controlled intervention trials have recently tested the effect of DR on metabolic health and age-related markers in nonobese men and women. The 1-year Washington University CALERIE (Comprehensive Assessment of Long-term Effects of Reducing Intake of Energy) trial demonstrated that a 14% DR and a 14% increase in energy expenditure through endurance exercise reduced visceral fat mass by ~35% and improved lipid profile, glucose tolerance, and insulin action, and reduced inflammation (Fontana *et al*., 2007b; Weiss *et al*., 2006). Results from the 6-month Pennington Center CALERIE trial show that a 13% DR decreased visceral fat mass, metabolic rate, body temperature, insulin resistance, and oxidative stress (Heilbronn *et al*., 2006). Observational data from healthy individuals practicing long-term DR with adequate nutrition (at least 100% of the RDI for each nutrient) show that even in these lean participants (mean BMI 19.6 kg m^−2^; ~12% body fat), DR induces beneficial changes against aging-related pathologies. In these individuals, DR caused rapid and profound improvements in all the major risk factors for CVD, including total cholesterol–HDL ratio (2.6 ± 0.5), triglycerides (48 ± 15 mg dL^−1^), fasting glucose (81 ± 7 mg dL^−1^), systolic (99 ± 10 mmHg) and diastolic (61 ± 6 mmHg) blood pressure, inflammatory markers (C-reactive protein, 0.3 ± 0.2 mg L^−^), and intima-media thickness (0.5 ± 0.1 mm) of the carotid arteries (Fontana *et al*., 2004; Holloszy & Fontana, 2007). This ‘super-physiological’ cardiometabolic profile in the CR practitioners most likely will result in a very-low lifetime risk of developing CVD and in a markedly longer median survival as suggested by longitudinal data of the Framingham Heart Study (Lloyd-Jones *et al*., 2006). Not only did DR in these lean individuals result in an optimal cardiovascular profile, but two markers of cardiovascular aging (i.e., left ventricular diastolic function and heart rate variability) were also significantly improved, consistent with the same adaptations seen in DR rodents (Meyer *et al*., 2006; Stein *et al*., 2012). Moreover, several metabolic and hormonal factors that have been associated with increased cancer risk (i.e., abdominal adiposity, insulin, testosterone, estradiol, leptin, and inflammatory cytokines) were all significantly lower in the DR group than in the nonobese controls (Longo & Fontana, 2010). Finally, the beneficial health effects of chronic DR are further supported by data collected from inhabitants of Okinawa Prefecture, Japan, who until 1960 had an average caloric intake 40% lower than US residents, a mean BMI of ~21 kg m^−2^, 80% lower rates of CVD and cancer mortality than the average US population, and one of the highest proportions of centenarians in the world (Willcox *et al*., 2007).

### Metabolic and molecular mechanisms underlying longevity and healthy aging

**Figure 5:**
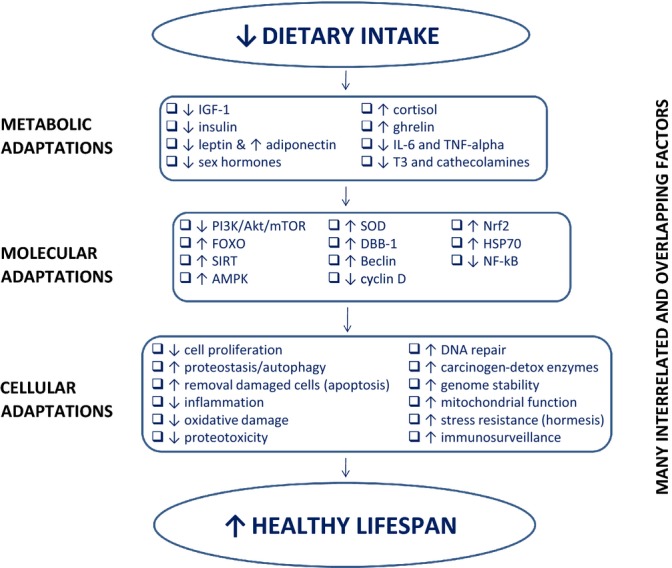
Dietary restriction (DR)-induced metabolic, molecular, and cellular adaptations. A chronic negative energy balance induced by dietary restriction triggers multiple metabolic adaptations including a reduction in circulating levels of growth factors (i.e., IGF-1), anabolic hormones (e.g., insulin, testosterone, estradiol, leptin), inflammatory cytokines (e.g., IL-6 and TNF-alpha), and key hormones that regulate thermogenesis and cellular metabolism (e.g., triiodothyronine (T3), cathecolamines), and an increase in hormones that suppress inflammation (e.g., cortisol, ghrelin, and adiponectin). Concomitantly, several key molecular adaptations take place, including a down-regulation of the insulin/IGF pathway (i.e., PI3K/Akt/mTOR), and an upregulation of two energy-sensing pathways (i.e., SIRT and AMPK) which activates FOXO. The activation of FOXO in turn modifies several ‘longevity genes,’ including up-regulation of antioxidant genes (e.g., SOD2), DNA repair genes (e.g., DDB1), autophagy genes (e.g., beclin), and a down-regulation of genes that control cell proliferation (e.g., cyclin D). DR induces also a down-regulation of inflammatory pathways (e.g., NFkB), and an up-regulation of genes that enhance protection against molecular damage (e.g., Nrf2, HSP70). These DR-induced metabolic and molecular modifications are responsible for several important cellular adaptations, that antagonize the accumulation of macromolecular damage, abnormal cell proliferation, and cellular senescence (Martin *et al*., 2006; Fontana *et al*., 2007a,b; Fontana *et al*., 2010; Guarente, 2013; Selman & Withers, 2011; Chen *et al*., 2010).

The exact molecular mechanisms underlying the beneficial effect of DR and the detrimental effects of a sustained positive energy balance leading to excessive adiposity remain unknown. However, data from dietary, genetic, and pharmacological studies in animal models of longevity and accelerated aging indicate that metabolism plays a crucial role in regulating health and survival (Kenyon, 2005; Russell & Kahn, 2007; Buffenstein & Pinto, 2009; Fontana *et al*., 2010; Selman & Withers, 2011). As shown in Fig. 5, many DR-induced interrelated and overlapping metabolic, molecular and cellular adaptations have been proposed to play a role in extending healthspan and lifespan (reviewed in-depth by Fontana & Klein, 2007; Chen *et al*., 2010; Fontana *et al*., 2010; Kourtis & Tavernarakis, 2011; Guarente, 2013; López-Otín *et al*., 2013). In particular, down-regulation of the key cellular nutrient-sensing pathways (e.g., the IGF/insulin/Akt/FOXO/mTOR pathway) has been shown to reduce disease risk and increase healthy survival in experimental animals (Fontana *et al*., 2010; Johnson *et al*., 2013). Consistently, several functional mutations of the human IGF-IR and FOXO3a gene, which results in down-regulation of the insulin/IGF signaling pathway, have been associated with extreme longevity in humans (Bonafè *et al*., 2003; Suh *et al*., 2008; Willcox *et al*., 2008; Flachsbart *et al*., 2009). Excessive intake of energy and essential amino acids stimulates this pathway, whereas calorie and protein restriction inhibits its activity (Giovannucci *et al*., 2003; Fontana *et al*., 2008, 2010; Efeyan *et al*., 2012; Mercken *et al*., 2013). Other metabolic factors that have been implicated in the pathogenesis of aging, but also frailty, and involuntary weight loss in both experimental studies of both animals and humans are inflammation, increased cortisol, and reduced levels of sex hormones and triidothyronine (Clegg *et al*., 2013). Although we do not yet know whether chronic inflammation is causally linked to the reduction in IGF-1, testosterone, and triidothyronine in elderly frail individuals, all of these factors are strong predictors of multidimensional impairment and mortality. Interestingly, however, lower IGF-1, triidothyronine and testosterone levels, and increased cortisol concentrations are also typical adaptations induced by DR in rodents (who live long, healthy lives) as well as humans (Weindruch & Walford, 1988; Masoro, 2005; Fontana & Klein, 2007). One of the main differences is that, unlike in elderly and frail animals and humans (Fontana *et al*., 2013), inflammation is extremely low in lean DR rodents and humans (Weindruch & Walford, 1988; Fontana & Klein, 2007; Meyer *et al*., 2006; Fontana *et al*., 2004), and the partial inhibition of these anabolic and mitogenic pathways (which when stimulated promote cancer and senescence) is driven by a reallocation of metabolic resources from growth to somatic maintenance and stress resistance (Fontana *et al*., 2010).

Unfortunately, only a small fraction of the US population (~7%) is both normal weight and practicing a healthy lifestyle (i.e., not smoking, exercising regularly, and eating a healthy diet) (Stampfer *et al*., 2000). Therefore, the increasingly U-shaped relationship between BMI and mortality with advancing age is probably the result of a diet-induced overstimulation of several mitogenic and inflammatory pathways starting early in life, which accelerates the accumulation of unrepaired cellular damage and senescent cells, activates the DNA damage response, inhibits the function of tissue stem cells, and promotes involuntary weight loss, organ dysfunction, and frailty (Bartke *et al*., 2002; Herbig *et al*., 2006; Schumacher *et al*., 2008; Fontana *et al*., 2010; Conboy & Rando, 2012; Campisi, 2013; López-Otín *et al*., 2013).

### Clinical and public health implications

The long-standing controversy regarding the relationship between BMI and mortality was heightened by a recent meta-analysis by Flegal *et al*. (Flegal *et al*., 2013). However, this study was fraught with methodological problems, which led to misleading conclusion that being overweight reduced mortality. A large body of epidemiological, clinical, and basic science evidence indicates that lean people who maintain a healthy body weight through a healthy diet and being physically active have healthier metabolic profiles, a lower risk of developing chronic disease, and a lower risk of mortality. In contrast, sedentary individuals eating a typical Western diet with a BMI < 25 who experience unintentional weight loss due to occult or preexisting chronic diseases or aging, coupled with increased levels of cortisol and inflammation, and low serum IGF-1 and/or testosterone concentrations may have an increased risk of debilitating chronic disease, frailty, and mortality. Undoubtedly, prevention strategies to maintain a healthy body weight and an optimal metabolic profile must start early, because even one major cardiovascular risk factor at 50 years of age considerably increases the risk of developing CVD and substantially shortens lifespan (Lloyd-Jones *et al*., 2006).

There is ample convincing evidence that overweight and obesity exert adverse influences on health and life expectancy to justify public health campaigns to prevent excessive weight gain and to promote the maintenance of a healthy weight throughout the lifespan. These data also justify the current BMI cutpoints for normal weight, overweight, and obesity. These standards should be primarily based on data that examine the relationships between BMI and chronic disease incidence rather than mortality, because mortality studies are highly susceptible to the aforementioned methodological problems. In addition to assessing BMI, it is important to monitor weight change after age 20 and changes in waist circumference, both of which are significant predictors of morbidity and mortality independent of current BMI. BMI should serve as a guidepost for health through life stages, and the prevention of weight gain is more important than weight loss because once an individual becomes obese, it is very difficult to achieve long-term weight loss and maintenance. In clinical settings, one should be sensible in the application of BMI cutpoints to individual patients, because not all patients who are modestly overweight need to lose weight, such as physically active athletes or body builders (Kalkhoff & Ferrou, 1971). In addition to assessing body weight and waist circumference, a metabolic and hormonal profiling is necessary to evaluate an individual’s overall risk of chronic diseases, frailty, and mortality.

Recently, the metabolically healthy but obese phenotype (overweight and obese adults who have no sign of metabolic disorders) has received much attention (Pataky *et al*., 2010; Stefan *et al*., 2013). However, this state is likely to be transitory for most individuals, especially for those who are not following a healthy diet nor exercising regularly (Appleton *et al*., 2013). On the other end of the spectrum, many individuals are normal weight but have an increased risk of metabolic diseases due to increased visceral adiposity. This ‘normal-weight metabolically obese’ phenotype, which is particularly common in Asian populations, deserves more clinical and public health attention (Chan *et al*., 2009). More studies are needed to identify more precisely the optimal body weight and metabolic/hormonal profile associated with successful aging and healthy longevity, which may vary based on age, sex, and genetic background. The best estimates of the impact of obesity on mortality should derive from cohort studies with large sample sizes and long follow-up periods from midlife or earlier.

## Conclusions

The continuing controversy regarding overweight and mortality has caused a great deal of confusion not only among the general public but also among health professionals. This controversy underscores the many methodological challenges in analyses of the relationship between BMI and mortality, including reverse causation, confounding by smoking, effect modification by age, and imperfect measures of adiposity. However, evidence for the adverse impact of overweight and moderate obesity on chronic disease incidence is overwhelming and indisputable. In addition, mounting evidence indicates that being overweight significantly reduces the probability of healthy aging. Many well-conducted studies in large cohorts have shown that being overweight does increase the risk of premature mortality. In these studies, after accounting for residual confounding by smoking and reverse causation, the lowest mortality is associated with a BMI < 25 kg m^−2^. The optimal BMI for most healthy middle-aged nonsmokers is likely to be in the lower and middle part of the normal range. The range of BMI (<25) that has been generally associated with desirable metabolic health and successful aging is supported by abundant data from DR studies in animal models and humans regarding metabolic parameters, disease risk, and longevity.
